# Antioxidant and Immunity Activities of Fufang Kushen Injection Liquid

**DOI:** 10.3390/molecules17066481

**Published:** 2012-05-30

**Authors:** Shen-Kang Zhou, Rui-Li Zhang, Yun-Feng Xu, Tie-Nan Bi

**Affiliations:** Department of Gastrointestinal Surgery, Taizhou Hospital of Zhejiang Province, Wenzhou Medical College, Linhai 318000, Zhejiang, China; E-Mails: skzhouwztz@163.com (S.-K.Z.); rlzhangwztz@163.com (R.-L.Z.); yfxuwztz@163.com (Y.-F.X.)

**Keywords:** FFKSIL, GSH-Px, antioxidant, immunity, Il-2, IgA, TNF-α

## Abstract

We investigated the effects of Fufang Kushen Injection Liquid (FFKSIL) on gastric immunity and oxidant-antioxidant status during *N*-methyl-*N′-*nitro-*N*-nitroso-guanidine (MNNG)-induced gastric carcinogenesis. The extent of lipid peroxidation and the levels of reduced glutathione (GSH) and activities of the GSH-dependent enzymes superoxide dismutase (SOD), catalase (CAT) and glutathione peroxidase (GSH-Px) were used to monitor the peroxidative balance. Enhanced lipid peroxidation in the gastric cancer animals was accompanied by significant decreases in the activities of GSH, GPx, GST and GR. Administration of FFKSIL significantly enhanced serum IgA, IgG, IgM, IL-2, IL-4 and IL-10 levels, decreased serum IL-6 and TNF-α levels, lowered the levels of lipid peroxides and enhanced GSH levels and activities of GSH-dependent enzymes. Our results suggest that FFKSIL blocks experimental gastric carcinogenesis by protecting against carcinogen-induced oxidative damage and improving immunity activity.

## 1. Introduction

Gastric cancer is one of the most frequent malignant neoplasms in the World, though the mortality rates vary in different countries and races. In 1991, Takahashi *et al*. [1] examined the effects of sodium chloride on lipid peroxidation levels in rat gastric mucosa. They concluded that administration of sodium chloride is associated with enhanced lipid peroxidation in gastric mucosa. Lipid peroxidation has been widely investigated as a possible mediator of various pathological and physiological process [2].

Reactive oxygen species-mediated tissue injury is a final common pathway for a myriad of disease processes. The body is continuously exposed to free radicals and ROS, from both external sources (sunlight, other forms of radiation, pollution) and generated endogenously. Oxidative stress can cause cancer [3], and it has been reported that the gastrointestinal tract is thought to be the major site for oxidant production but is also a source of antioxidants [4]. Oxidative stress can modulate the apoptotic program [5] and could lead to gastric cancer [6].

The agent *N*-methyl-*N*-nitrosourea (MNU) is a direct acting carcinogen, inducing tumors in several species in a variety of organs, including the central nervous system, stomach, intestine, kidney, and skin [7,8,9,10]. Treatment of MNU in the drinking water for 25–42 weeks selectively induced glandular gastric carcinoma in male F344 rats [11,12] and a mouse model for gastric carcinogenesis was recently established by using oral administration of MNU [13]. These animal models have been widely used not only for investigating the pathogenesis of gastric carcinogenesis but also for identifying possible tumor promoters and chemopreventive agents [14,15].

The compound recipe Radix Sophorae Flavescentis (Kushen) for injection (CRS) is a freeze dried powder prepared from an extract of Sophora flavescentis and Smilacis rhizoma. Sophorae flavescentis is the dry root of the leguminosae plant *Sophora flavescens* Ait. It is described in “Shennong’s Herbal” (a famous ancient chinese medicine book). Modern pharmacological research has shown that Sophora flavescentis has an anti-tumor effect. It is reported recently that the Fufang Kushen Injection Liquid (FFKSIL) possesses cytotoxic effects against gastric cancer BGC-823 cell lines proliferation [16]. In addition, it is reported that combination of FFKSIL and chemotherapy has been applied to clinical therapy of human gastric cancer [17]. Our objectives were to evaluate the antioxidant and immunity activity of FFKSIL in MNNG-induced gastric cancer rats. 

## 2. Results and Discussion

Immunoglobulins (Ig) play a major role in adaptive immunity. Complete or near complete loss of certain species of immunoglobulin often occurs in some primary immunodeficiency syndromes. This is known to increase the risk of certain types of infection, depending on which immunoglobulin species is deficient. Secondary or acquired immunodeficiency is a common clinical condition resulting from a wide range of primary causes, including organ transplant, leukemic disorders that affect B-cells, and the administration of certain drugs [18,19]. 

Table 1 shows that in the present study the serum IgA, IgM, and IgG levels were significantly lower in the model control (MC) group than in the normal control (NC) group (*p* < 0.05). This suggests that immunity function has been decreased in gastric cancer animals. The FFKSIL treatment (2, 3, 4 mL/kg b.w.) dose-dependently markedly enhanced the serum IgA, IgM, and IgG levels in the FFKSIL groups compared to group MC. Our result showed that FFKSIL treatment could enhance immunity function in gastric cancer animals.

Interleukin-2 (IL-2) is a potent T cell growth factor that has been used clinically to augment T cell-mediated immune responses, and as a vaccination adjuvant [20,21,22,23,24,25]. Interleukin-4 (IL-4) plays an important role in regulating the immune response of B cells, T cells, and macrophages against infections and malignant cells [26,27,28]. Interleukin-10 (IL-10) is a multifunctional cytokine with both immunosuppressive and anti-angiogenic functions and consequently has both tumor-promoting and tumor-inhibiting properties. Raised levels of serum and peritumoral IL-10 production have been reported in many malignancies [29,30,31], including lung cancer [32], which have been interpreted in support of a role for IL-10 in tumor escape from the immune response. Table 2 shows that the serum IL-2, IL-4, and IL-10 levels were significantly lower in the MC group than in the NC group (*p* < 0.05). The three doses of FFKSIL treatment dose dependently markedly enhanced the serum IL-2, IL-4, and IL-10 levels in the FFKSIL groups compared to group MC.

Interleukin-6 (IL-6) is a pleiotropic inflammatory cytokine. First discovered as a B-cell growth factor, it is synthesized by many cell types, including T-cells, macrophages and stromal cells, in response to stimulation from tumour necrosis factor-alpha (TNF-a) and interleukin-1 (IL-1) [33,34]. The activation of the IL-6 complex activates Janus kinases (JAK), signal transducers and activators of transcription (STATs), which regulate cell proliferation and apoptosis [34,35].

Tumor necrosis factor-α (TNF-α), a multifunctional cytokine, is involved in the promotion of inflammatory responses and plays a critical role in the pathogenesis of inflammatory, autoimmune, and malignant diseases [36,37]. Initially proposed to have anti-carcinogenic effects [38], TNF was later shown to be tumorigenic in both in vitro and *in vivo* studies. High plasma TNF levels in cancer patients have been associated with a poor disease outcome [39,40]. TNF is also a key angiogenic molecule that may promote angiogenesis directly, by stimulating endothelial cell proliferation, and indirectly, by modulating expressions of other proangiogenic factors [41,42,43]. Moreover, TNF is known to induce expression of adhesion molecules, despite being involved in the increased motility and invasive/metastatic behaviour of tumor cells [44].

In the MC group, the levels of serum IL-6 and TNF-α was higher than those in NC group (*p* < 0.05) (Table 3). Treatment with FFKSIL dose-dependently significantly decreased the concentration of IL-6 and TNF-α (*p* < 0.05). When anti-inflammatory cytokines (IL-2, IL-4, IL-10) was released less, proinflammatory cytokines (IL-6 and TNF-α) was excessively released. As a result, this would promote excessive inflammatory response and induce systemic inflammatory response syndrome (SIRS) and multiple organ dysfunction syndrome (MODS). Our result showed that FFKSIL treatment could improve immunity activities by stimulating anti-inflammatory cytokines (IL-2, IL-4, IL-10) release and inhibiting proinflammatory cytokines (IL-6 and TNF-α) production in gastric cancer rats.

Gastric cell and tissue injury associated with acute and chronic inflammation is due to the toxicity of ROS generated in stomach [45]. It has been widely accepted that a large number of free radicals is generated in the peptic ulcer and gastritis, but its mechanism is unclear. Oxygen-derived free radicals play an important role in the pathogenesis of injuries of the digestive system [46,47,48]. In addition, the involvement of oxygen-derived free radicals in the pathogenesis of ischaemic injury of gastrointestinal mucosa and in other models of mucosal damage induced by non-steroidal anti-inflammatory drugs, ethanol, and *H. pylori* is well established [49].

Our work showed that in the MC group, the level of malondialdehyde (MDA) was higher, whereas concentration of glutathione (GSH) was lower in the serum of the NC group (*p* < 0.05) compared to NC group (Table 4). Treatment with FFKSIL dose-dependently significantly decreased the concentration of MDA and significantly increased GSH in serum and gastric mucosa of rats in FFKSIL groups compared to MC group (*p* < 0.05). 

In the MC group, the activities of serum and gastric mucosa superoxide dismutase (SOD), catalase (CAT) and glutathione peroxidase (GSH-Px) were lower than those in NC group (*p* < 0.05) (Table 5). Treatment with FFKSIL dose-dependently significantly increased the activities of serum and gastric mucosa SOD, CAT and GSH-Px (*p* < 0.05). These results indicate that FFKSIL ameliorates oxidative stress in gastric cancer rats. 

## 3. Experimental

### 3.1. Material

Fufang Kushen injection liquid was purchased from Shanxi Zhendong Pharmacy Ltd. (Shanxi, China).

### 3.2. Animals and Grouping

Male Wistar albino rats (180–210 g) were used as experimental models with the approval of the Experimental Animal Ethics Committee of China. They were housed in standard polypropylene cages, kept under ambient temperature (25–30 °C) and relative humidity of 60–70% in a 12 h light–dark cycle. The animals were provided with food and water *ad libitum*. The animals were randomised into experimental and control groups and divided into four groups of ten animals each. Rats in group 2 (MC) were given MNNG (150 mg/kg body weight) by intragastric intubation three times with a gap of two weeks in between the treatments [50]. Rats in groups 2, 3, 4 were administered MNNG as in group 1, and in addition received Fufang Kushen injection liquid (2 mL/kg, 3 mL/kg or 4 mL/kg body weight) once per day starting on the day following the first exposure to MNNG and continued until the end of the experimental period. Another ten rats (group 1) were served as normal control (NC) and didn’t receive MNNG (150 mg/kg body weight) and Fufang Kushen injection liquid. The experiment lasted for two weeks. At the end of the feeding period, the animals were sacrificed and blood sample collected for analysis. The stomachs were rapidly excised, weighed, and immediately frozen in liquid nitrogen and stored at −70 °C until use. Homogenates were centrifuged at 1,000 × g for 10 min at 4 °C, and the supernatants were used for the determination of enzymatic activities.

### 3.3. Serum Ig Levels

The IgA, IgG and IgM levels in the serum were determined by radial immunodiffusion (RID) plates (The Binding Site Ltd., Birmingham, UK), which contained anti-serum specific to the antigen. The recommended amount of serum was put into the wells of plates and incubated for 72–96 h at room temperature. The diameter of the precipitation ring was then measured and the concentrations of Igs were determined by using standard nomograms.

### 3.4. IL-2, IL-4, IL-10, IL-6, TNF-α Levels

IL-2, IL-4, IL-10, IL-6 and TNF-α levels were measured using commercially available ELISA kits. All procedures were performed according to manufacturer’s instructions. 

### 3.5. Measurement of Antioxidant Enzymes Activities

Samples stored at −80 °C was used to measure the lipid peroxidation inhibition. Thiobarbituric acid reactive substances (TBARS) content of the urine samples was determined by the method of Draper *et al*. [51] with slight modifications. Serum sample (1 mL) was mixed with 30% trichloroacetic acid (1 mL) and 0.67% thiobarbituric acid (1 mL) in the presence of 200 mM butylated hydroxytoluene (0.1 mL). The mixture was heated at 90 °C for 45 min in a tightly closed tube in boiling water. After cooling, the tubes were centrifuged at 3,000 rpm for 10 min at 5 °C and the absorbance of the supernatant was measured at 532 nm at room temperature using a Perkin–Elmer Lambda 45 spectrophotometer. The amount of TBARS produced was calculated using 1,1,3,3-tetra- ethoxypropane as a standard. Lipid peroxidation was expressed in nmoles of TBARS formed per mL of the sample.

The samples were homogenised with a solution of 0.5 M perchloric acid containing 5 mM EDTA and 0.06% (w/v) bipiridine. The total GSH was determined by measuring the reduction of 5,50-dithio-2-nitrobenzoic acid by NADPH in the presence of glutathione reductase according to Murphy and Keher [52]. Values were determined by comparing the reduction rate against a standard curve of glutathione. Oxidised glutathione (GSSG) was determined before the addition of glutathione reductase. 

SOD activity was determined as described by McCord and Fridovich [53]. The assays were carried out using various amounts of tissue sample in a reaction containing 0.1 M sodium phosphate buffer pH 7.8, 0.3 mM hypoxanthine, 7.5 mM cytochrome c (cyt c). The reaction was started by adding 5 U of xanthine oxidase in a final volume of 1 mL. Cyt c reduction was observed for 60 s at 550 nm in a Hitachi model U-2000 dual beam spectrophotometer equipped with a water-jacketed cell holder. The results of this enzymatic assay were shown in units of SOD (U mL^−1^ or mg^−1^) where one unit of SOD is defined as the amount able to inhibit 50% cyt c reduction at 25 °C.

Glutathione peroxidase (GSH-Px) activity was analyzed by a spectrophotometric assay. A reaction mixture consisting of 1 mL of 0.4 M phosphate buffer (pH 7.0) containing 0.4 mM EDTA, 1 mL of 5 mM NaN_3_, 1 mL of 4 mM GSH, and 0.2 mL of supernatant was preincubated at 37 °C for 5 min. Then 1 mL of 4 mM H_2_O_2_ was added and incubated at 37 °C for further 5 min. The excess amount of GSH was quantified by the DTNB method as described by Sharma and Gupta [54]. One unit of GSH-Px is defined as the amount of enzyme required to oxidize 1 nmol GSH/min.

Catalase (CAT) activity was assayed following the method of Sinha [55]. The reaction mixture consisted of 150 μL phosphate buffer (0.01 M, pH 7.0), 100 μL supernatant. Reaction was started by adding 250 μL H_2_O_2_ 0.16 M, incubated at 37 °C for 1 min and reaction was stopped by the addition of 1.0 mL of dichromate:acetic acid reagent. The tubes were immediately kept in a boiling water bath for 15 min and the green colour developed during the reaction was read at 570 nm on a spectrophotometer. Control tubes, devoid of enzyme, were also processed in parallel.

### 3.6. Statistical Analysis

All results are expressed as mean ± S.E.M. Statistical analyses were performed using one-way analysis of variance (ANOVA). Significant differences were determined by Tukey’s post hoc test. F values for which *p* < 0.05 were regarded as statistically significant. 

## 4. Conclusions

Our data suggest that FFKSIL shows anti-inflammatory effects in *in vivo*. The anti-inflammatory effects of FFKSIL may be related to IgA, IgM, IgG, IL-6 and TNF-α production and IL-2, IL-4 and IL-10 reduction in gastric cancer rats. The antioxidant effects of FFKSIL can be due to increase in the activities of antioxidant enzymes and its effects on radicals scavenging. Therefore, we suggest that FFKSIL possess antioxidants and anti-inflammatory activity *in vivo*.

## Figures and Tables

**Table 1:** Effect of FFKSIL treatment on serum IgA, IgG and IgM.

Group	IgA (mg/L)	IgM (mg/L)	IgG (mg/L)
NC	147 ± 13	1683 ± 121	23156 ± 2085
MC	58 ± 4 ^b^	1229 ± 112 ^b^	19352 ± 1593 ^b^
FFKSIL (2 mL/kg b.w.)	86 ± 12 ^d^	1384 ± 141 ^c^	20461 ± 1837
FFKSIL (3 mL/kg b.w.)	111 ± 14 ^d^	1433 ± 137 ^d^	21942 ± 1769 ^c^
FFKSIL (4 mL/kg b.w.)	137 ± 14 ^d^	1575 ± 139 ^d^	22471 ± 2011 ^d^

^b^ *p* < 0.01, compared with group NC; ^c^ *p* < 0.05, ^d^ *p* < 0.01, compared with group MC; Body weight (b.w.).

**Table 2:** Effect of FFKSIL treatment on serum IL-2, IL-4 and IL-10 levels.

Group	IL-2 (ng/mL)	IL-4 (ng/mL)	IL-10 (ng/mL)
NC	6.07 ± 0.45	20.85 ± 1.83	91.48 ± 5.09
MC	4.39 ± 0.28 ^b^	14.73 ± 1.11 ^b^	63.51 ± 3.17 ^b^
FFKSIL (2 mL/kg b.w.)	4.98 ± 0.30	17.49 ± 1.32 ^d^	74.29 ± 3.52 ^c^
FFKSIL (3 mL/kg b.w.)	5.73 ± 0.34 ^c^	18.88 ± 1.29 ^d^	82.91 ± 3.77 ^d^
FFKSIL (4 mL/kg b.w.)	6.11 ± 0.29 ^d^	19.79 ± 1.42 ^d^	89.93 ± 4.29 ^d^

^b^ *p* < 0.01, compared with group NC; ^c^ *p* < 0.05, ^d^ *p* < 0.01, compared with group MC.

**Table 3:** Effect of FFKSIL treatment on serum IL-6 and TNF-α levels.

Group	IL-6 (ng/mL)	TNF-α (ng/mL)
NC	70.54 ± 4.86	2.45 ± 0.18
MC	89.69 ± 5.05 ^b^	3.87 ± 0.19 ^b^
FFKSIL (2 mL/kg b.w.)	82.15 ± 4.97 ^c^	3.28 ± 0.22
FFKSIL (3 mL/kg b.w.)	76.09 ± 4.82 ^c^	2.96 ± 0.21 ^d^
FFKSIL (4 mL/kg b.w.)	72.13 ± 3.98 ^d^	2.57 ± 0.17 ^d^

^b^ *p* < 0.01, compared with group NC; ^c^ *p* < 0.05, ^d^ *p* < 0.01, compared with group MC.

**Table 4:** Effect of FFKSIL treatment on serum and gastric mucosa MDA and GSH levels.

Group	MDA		GSH	
	Serum	Gastric mucosa	Serum	Gastric mucosa
NC	4.83 ± 0.29	3.79 ± 0.21	231.76 ± 18.32	121.04 ± 9.92
MC	8.49 ± 0.53 ^b^	7.92 ± 0.53 ^b^	158.24 ± 12.14 ^b^	68.39 ± 4.28 ^b^
FFKSIL (2 mL/kg b.w.)	7.03 ± 0.41 ^c^	5.82 ± 0.25 ^d^	188.26 ± 11.95 ^d^	89.37 ± 5.63 ^d^
FFKSIL (3 mL/kg b.w.)	5.82 ± 0.22 ^d^	5.11 ± 0.31 ^d^	204.73 ± 13.29 ^d^	108.24 ± 7.04 ^d^
FFKSIL (4 mL/kg b.w.)	4.71 ± 0.19 ^d^	4.27 ± 0.24 ^d^	223.18 ± 14.82 ^d^	119.32 ± 8.94 ^d^

^b^ *p* < 0.01, compared with group NC; ^c^ *p* < 0.05, ^d^ *p* < 0.01, compared with group MC.

**Table 5:** Effect of FFKSIL treatment on serum and gastric mucosa SOD, CAT, GSH-Px activities.

Group	SOD		CAT		GSH-Px	
	Serum	Gastric mucosa	Serum	Gastric mucosa	Serum	Gastric mucosa
NC	201.5 ± 18.38	174.3 ± 12.69	45.26 ± 2.68	38.29 ± 1.58	52.16 ± 3.52	47.73 ± 2.68
MC	137.2 ± 11.32 ^b^	103.2 ± 8.49 ^b^	23.18 ± 1.65 ^b^	17.04 ± 1.21 ^b^	28.38 ± 1.69 ^b^	25.81 ± 2.01 ^b^
FFKSIL (2 mL/kg b.w.)	170.5 ± 10.63 ^d^	139.5 ± 8.84 ^d^	33.29 ± 1.48 ^d^	24.72 ± 1.59 ^d^	39.91 ± 1.93 ^d^	31.54 ± 1.82 ^d^
FFKSIL (3 mL/kg b.w.)	188.3 ± 13.29 ^d^	158.3 ± 10.42 ^d^	39.17 ± 1.77 ^d^	30.14 ± 1.99 ^d^	44.73 ± 2.64 ^d^	39.54 ± 1.95 ^d^
FFKSIL (4 mL/kg b.w.)	197.2 ± 13.09 ^d^	177.2 ± 11.43 ^d^	48.32 ± 2.07 ^d^	39.12 ± 2.14 ^d^	49.81 ± 3.32 ^d^	49.03 ± 2.49 ^d^

^b^ *p* < 0.01, compared with group NC; ^d^ *p* < 0.01, compared with group MC.
